# Advanced Signal Processing Methods for Biomedical Imaging

**DOI:** 10.1155/2013/696878

**Published:** 2013-01-30

**Authors:** Juan Ruiz-Alzola, Carlos Alberola-López, Carl-Fredrik Westin

**Affiliations:** ^1^Department of Signals and Communications, University of Las Palmas de Gran Canaria, 35017 Las Palmas de Gran Canaria, Spain; ^2^Canary Islands Agency for Reseach, Innovation and Information Society, 35003 Las Palmas de Gran Canaria, Spain; ^3^Image Processing Laboratory, ETSI Telecomunicación, University of Valladolid, 47011 Valladolid, Spain; ^4^Laboratory for Mathematics in Imaging, Brigham and Women's Hospital, Harvard Medical School, Boston, MA 02115, USA

Signal processing is a long-established engineering field with broad applications in so different arenas such as multimedia (audio, still image, video, graphics, etc.), coding, communications, seismology, astronomy, biomedicine, artificial intelligence, and econometrics, to only name some of them. Signal processing stems from cross-fertilized grounds including mathematical analysis, algebra, numerical analysis, probability, statistics, information theory, discrete mathematics, cybernetics, and computer science. Advances in signal processing have frequently been pulled by needs in specific application domains, so that they are readily incorporated to the signal processing knowledge base for convenient use in distant areas of application. 

Signal and image processing is ubiquitous in modern biomedical imaging, as it provides essential techniques for image construction, enhancement, coding, storage, transmission, analysis, understanding, and visualization from any of an increasing number of different multidimensional sensing modalities. As biomedical imaging is rapidly evolving, new and more powerful signal and image processing algorithms are required to meet the challenges imposed by modern huge multidimensional multimodal biomedical data, particularly in real clinical settings.

In this special issue, we present high-quality original research papers that address specific challenges for the biomedical imaging community and benefit of advanced and emerging methods in signal and image processing required for novel time-varying high-dimensional structural and functional biomedical imaging modalities. Interesting new ideas from other fields of application of signal and image processing have been highly welcome since their practical relevance for biomedical imaging is clearly motivated.

In particular, this special issue features original research for different imaging modalities, such as generalized diffusion tensor imaging (GDTI), conventional diffusion weighted MRI (DW-MRI), dynamic contrast enhanced MRI (DCE-MRI), functional MRI (fMRI), X-ray mammography and time-frequency representations (TFR) of electrophysiological data such as electroencephalography (EEG), magnetoencephalography (MEG), or local field potentials (LFP). The medical application areas include central nervous system fiber tractography, analysis of episodic memory and motor brain activity, analysis of neural activity patterns, and breast cancer screening and detection. As for the signal and image processing methods, a brief list of topics addressed in this special issue include higher-order tensors, signal estimation and classification, clustering, and multivariate statistics, to name a few. 

In “*Fast and analytical EAP approximation from a 4th-order tensor*,” A. Ghosha and R. Deriche deal with the complicated problem of inferring the microstructure of tissue or fiber bundles from DW-MRI. To this extent they propose a modified GDTI approach, where the higher-Order tensor (HOT) representation of the apparent diffusivity coefficient (ADC) is estimated, from the DW-MRI data, in such a way that they provide a closed-form approximation, using Hermite polynomials, to the ensemble average propagator (EAP) that overcomes the computational overload of other EAP estimation methods from GDTI. The EAP describes the probability of the diffusing particles, and, hence, the geometry of the EAP is a direct indicator of the microstructure of the underlying tissue or fiber bundles. They provide experimental results on the fiber bundles estimated from real and synthetic brain datasets.

In “*DCE-MRI and DWI integration for breast lesions assessment and heterogeneity quantification*,” C. A. Méndez et al. address the quantification of breast tumor heterogeneity using jointly DW-MRI and DCE-MRI, which provide a measure of cellularity and an indication of blood volume, flow, and vascular permeability, respectively. To that end, they first make an affine followed by an elastic registration of both datasets and approach the tissue segmentation by clustering using a dissimilarity-based representation (DBR) of the DCE curves and the ADC maps, followed by K-means. Statistical testing is carried out for the ADC maps of the resulting clusters. They provide experimental results from 21 patients with primary ductal carcinoma.

In “*The smoothing artifact of spatially constrained canonical correlation analysis in functional MRI*,” D. Cordes et al. address the problem of the spatial specificity of the activation signal in fMRI due to smoothing artifacts that arise in an extension of the canonical correlation analysis (CCA) termed constrained CCA (cCCA), which is used as a statistic in fMRI to test for functional brain activation in a specific neighborhood. In particular, they investigate in detail the smoothing artifact that is associated with each spatial constraint in cCCA and provide a novel approach in order to correct the measure of activation for the smoothing artifact within a Bayesian testing framework. They also provide experimental results on six real cases with six normal subjects carrying out certain motor and episodic memory tasks.

In “*A new GLLD operator for mass detection in digital mammograms*,” N. Gargouri et al. propose an extension to the local binary pattern (LBP) operator used for texture classification in order to overcome some limitations that arise from the fact that LBP uses local gray-level differences. In particular they introduce the gray level and local Difference (GLLD) representation that, in addition to differences, also uses gray levels and investigate the efficiency of the GLLD-based approach as a method of feature extraction. They tackle the detection of abnormal masses on breast X-ray images by performing a manual region of interest (ROI) delineation, followed by the GLLD representation and standard classification by k-NN, support vector machines, and multilayer perceptrons. Experimental results are from 1000 ROIs from the Digital Database for Screening Mammography.

Last but not least, in “*A flexible model for feature extraction from brain signals in the time-frequency domain*,” R. Heideklang and G. Ivanova provide a different perspective since they deal with electrophysiological data. Images arise from the time-frequency representation (TFR) of these nonstationary signals that are obtained in the paper from the smoothed pseudo-Wigner-Ville distribution. A common problem of TFRs from electrophysiological data is inter- and intrasubject variability of the features to be extracted (curves in the TFR), which is here addressed by proposing the smooth natural Gaussian extension (snaGe) model, characterized by a sequence of multivariate Gaussians located on a parametric curve described by a cubic B-spline. By combining cubic B-splines with the Gaussian shape, they obtain a “sufficiently smooth model which inherits both the splines' flexibility and the Gaussian standard model's robustness.” In order to fit the model to the data, after subsampling, an initial estimation of the initial parameters of the curve is obtained by dynamic programming, which is refined through iteration, and using Fréchet and other metrics for curve difference. The authors present results on real and synthetic EEG data, and they discuss the robustness and the sensitivity of the method to noise.

We hope that the readers enjoy this special issue.

*Juan Ruiz-Alzola* *Juan Ruiz-Alzola* *Carlos Alberola-López* *Carlos Alberola-López* *Carl-FredrikWestin* *Carl-FredrikWestin*

